# Treatment burden and health-related quality of life in adults with multiple conditions: a cross-sectional study of associated factors

**DOI:** 10.3389/fpubh.2026.1987212

**Published:** 2026-10-14

**Authors:** Zhaoxuan Wang, Kang Wang, Xin Zhang, Yelin Feng, Yuting Yang, Nannan Cheng, Xuan Chang, Wenqiang Yin, Zhongming Chen, Dongping Ma

**Affiliations:** 1 School of Management, Shandong Second Medical University, Weifang, Shandong, China; 2 Health Management Research Center, Shandong Second Medical University, Weifang, Shandong, China; 3 Department of Finance, Weifang People's Hospital, Weifang, Shandong, China; 4 Weifang Eye Hospital, Weifang, Shandong, China; 5 Shandong Provincial Hospital Affiliated to Shandong First Medical University, Jinan, Shandong, China

**Keywords:** EQ-5D-5L, health utility index, health-related quality of life, multimorbidity, primary care, treatment burden

## Abstract

**Background:**

Treatment burden and health-related quality of life (HRQoL) are closely interrelated patient-centered outcomes among people with multimorbidity, and several socioeconomic, clinical, and behavioral correlates of treatment burden have already been identified. However, the distribution and relative prominence of these characteristics may vary across regional socioeconomic and healthcare contexts. This study examined factors associated with moderate/high treatment burden among adults with multimorbidity in Shandong Province, China, and explored the relative importance of the analyzed factors using complementary random forest analysis.

**Methods:**

This secondary analysis used cross-sectional data from 765 adults with multimorbidity who participated in the Survey of Residents’ Health and Contracted Family Doctor Services in Shandong Province, China, in 2023. Treatment burden was assessed using the Chinese version of the Multimorbidity Treatment Burden Questionnaire (C-MTBQ), and HRQoL was assessed using the EuroQol Five-Dimension Five-Level Questionnaire (EQ-5D-5L). Participants were classified into no/low and moderate/high treatment burden groups. Univariable analyses and multivariable logistic regression were performed to identify factors independently associated with moderate/high treatment burden. A supplementary random forest analysis was conducted using the variables retained in the regression model to describe their relative importance within the selected predictor set. Model discrimination was summarized using the area under the receiver operating characteristic curve (AUC).

**Results:**

Overall, 65.8% of participants experienced moderate/high treatment burden. Lower health utility index, lower household income, higher annual household healthcare expenditure, having three chronic conditions, lower educational attainment, and lower frequency of physical activity were independently associated with moderate/high treatment burden (all *p* < 0.05). The random forest analysis yielded an AUC of 0.890. Among the included variables, health utility index showed the highest relative importance, followed by annual household income, annual household healthcare expenditure, physical activity, and number of chronic conditions.

**Conclusion:**

In this sample of adults with multimorbidity from Shandong Province, treatment burden was associated with HRQoL and several socioeconomic, clinical, and behavioral characteristics. These findings should be interpreted within the regional context of the study and should not be assumed to represent adults with multimorbidity across China.

## 1 Introduction

With the accelerating pace of population ageing, multimorbidity has emerged as a major global public health challenge (1). Multimorbidity is commonly defined as the coexistence of two or more chronic conditions in the same individual (2). Multimorbidity is associated not only with an increased risk of mortality but also with poorer quality of life and greater demand for healthcare resources (3). Globally, the prevalence of multimorbidity has increased substantially with population ageing, affecting approximately one-third of adults and more than half of older adults (4). In China, recent epidemiological studies have reported that the prevalence of multimorbidity among adults aged 60 years and above exceeds 40% (5), posing considerable challenges to healthcare systems and chronic disease management. As treatment regimens become increasingly complex, treatment burden has emerged as an important patient-centered outcome requiring greater attention (5). Population ageing has contributed to a growing prevalence of multimorbidity, particularly among older adults, placing increasing pressure on primary healthcare systems (1, 6, 7). Compared with individuals living with a single chronic condition, patients with multimorbidity are often required to manage long-term medication regimens, attend frequent medical appointments, undertake self-monitoring, and implement multiple lifestyle modifications, making disease management considerably more complex (8, 9). Consequently, these patients must bear not only higher financial costs but also substantial time, physical, and psychological demands associated with completing treatment-related tasks (10, 11). Against this background, treatment burden has attracted increasing attention in both clinical practice and health services research. Treatment burden refers to the workload imposed on patients by healthcare and self-management activities, together with the effects of this workload on their daily lives. It encompasses medication management, healthcare visits, self-monitoring, lifestyle modification, and financial and psychological pressures (12, 13). Previous studies have shown that a high treatment burden may reduce treatment adherence and health-related quality of life and may also contribute to poorer disease control and an increased risk of hospital readmission (14, 15).

Research on treatment burden has expanded substantially in recent years, particularly in relation to the development and validation of measurement instruments (16), the identification of associated factors (17), and the evaluation of intervention strategies (18). Previous studies have identified a range of socioeconomic, clinical, behavioral, and health-status characteristics associated with treatment burden among people with multimorbidity. HRQoL has also been consistently linked to treatment burden. Burden of Treatment Theory (BOTT) provides a useful conceptual perspective for understanding these relationships by emphasizing the balance between the workload imposed by treatment and self-management and the capacity available to patients to manage these demands (14). In this framework, socioeconomic and personal resources may influence patients’ capacity, whereas clinical demands contribute to treatment workload and HRQoL reflects patients’ perceived health and functional wellbeing. In the present study, BOTT was used as an interpretive framework rather than as a formal theory-testing model.

Building on existing evidence, this study examined factors associated with moderate/high treatment burden among adults with multimorbidity in Shandong Province, China, with particular attention to HRQoL assessed using the EQ-5D-5L health utility index. The regional rationale was to examine these established domains in a sample drawn from areas with different levels of socioeconomic development within the province, without assuming that the associations would differ fundamentally in direction from those reported elsewhere. Multivariable logistic regression was used to estimate adjusted associations, while complementary random forest analysis described the relative importance of variables retained in the regression model. The contribution of the study is therefore regional and integrative, bringing together HRQoL and socioeconomic, clinical, and behavioral characteristics within the same analytical framework. The random forest analysis was supplementary and was not intended as an independent feature-selection procedure or a validated clinical prediction model.

## 2 Methods

### 2.1 Study design and participants

This study was a secondary analysis of data from the Survey of Residents’ Health and Contracted Family Doctor Services, conducted in 2023 among general residents in Shandong Province, China. The original survey was organized by the research group led a corresponding author of the present manuscript, and primarily examined residents’ health, family doctor contracting, and service utilization. Its questionnaire included the complete Chinese version of the Multimorbidity Treatment Burden Questionnaire (C-MTBQ). Previous publications arising from the survey have examined family doctor service preferences and the relationship between multimorbidity and quality of life (19, 20). WY, ZC, and DM were responsible for questionnaire design in the original survey. ZW and NC contributed to the sampling design and selection of measurement instruments. The remaining authors did not participate in the original survey; their contributions related to the present study and preparation of the manuscript.

The present analysis focused on 765 adults with multimorbidity and examined treatment burden and its associations with health-related quality of life and sociodemographic, clinical, and behavioral characteristics. Participants were recruited using a multistage stratified sampling strategy designed to include areas with different levels of socioeconomic development within Shandong Province. At the first stage, three prefecture-level cities representing relatively high, medium, and low levels of economic development were included. Within each city, three counties, county-level cities, or districts were selected according to local socioeconomic conditions. Four townships or subdistricts were subsequently selected from each county-level area, followed by six villages or communities from each township or subdistrict. Finally, five to eight eligible residents were recruited from each selected village or community. The multistage design was intended to increase socioeconomic and geographic diversity within the study area rather than to establish national representativeness. Geographic and socioeconomic stratification was used to capture contextual variation across study areas, whereas demographic and clinical characteristics were assessed at the individual level. The original survey used different questionnaire versions. The present analysis drew on the version containing the complete Chinese version of the Multimorbidity Treatment Burden Questionnaire (C-MTBQ). For this version, 1,158 questionnaires were distributed and 1,148 were returned. After exclusion of 383 questionnaires that did not meet the eligibility criteria or data-quality requirements, 765 adults with multimorbidity were included in the present analysis (Figure 1).

**Figure 1:**
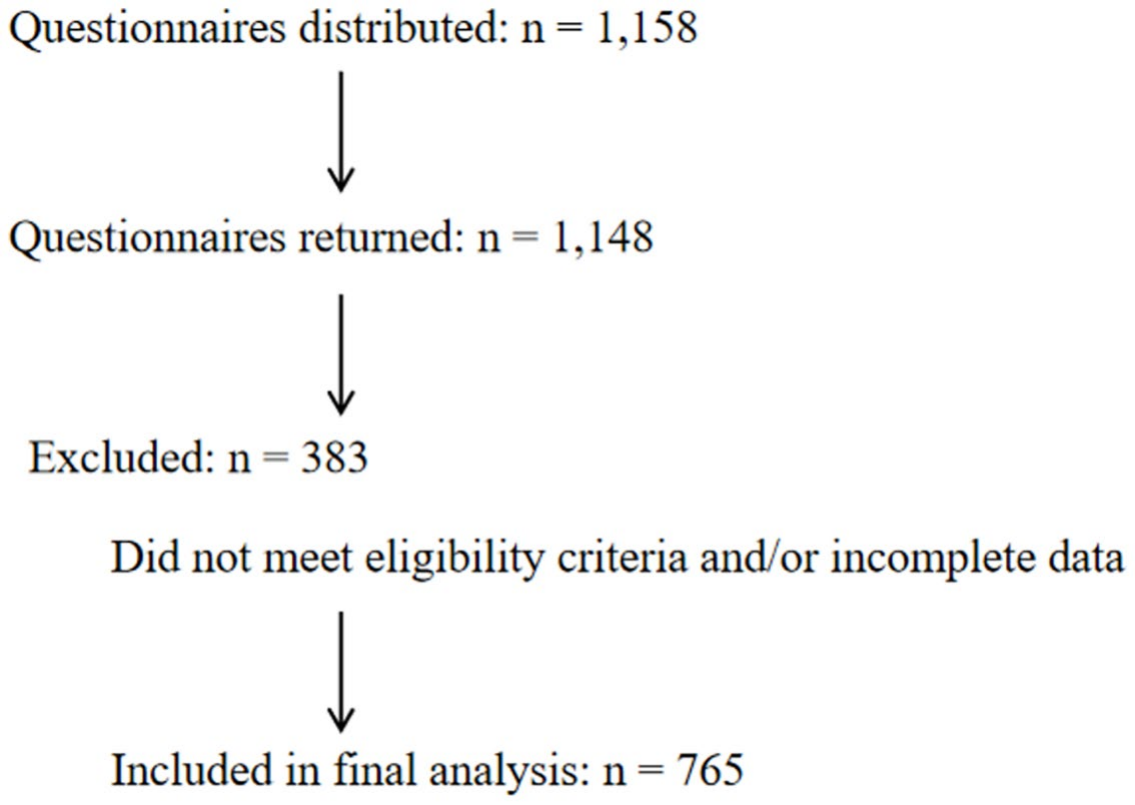
Flowchart of participant selection.

Participants were eligible if they: (1) were aged 18 years or older; (2) For the present study, multimorbidity was operationally defined as the coexistence of at least two of the 14 chronic conditions included in the original survey questionnaire, had been diagnosed with at least two of the 14 chronic conditions considered in this study, including hypertension, diabetes mellitus, coronary heart disease, stroke, cerebral infarction, cervical spondylosis, liver cirrhosis, rheumatoid arthritis, malignant tumors, lumbar muscle strain, scapulohumeral periarthritis, chronic gastroenteritis, chronic bronchitis, and hyperlipidemia. These conditions were based on the chronic-disease items included in the original questionnaire and were not selected specifically for the present analysis. The condition list was not designed to reproduce standardized multimorbidity lists such as those proposed by Barnett et al. or Fortin et al., which should be considered when comparing the present findings with other studies; (3) voluntarily agreed to participate in the study; and (4) Participants with severe psychiatric, intellectual, cognitive, or communication impairment that prevented them from understanding or completing the interviewer-administered questionnaire were excluded in accordance with the original survey protocol.

### 2.2 Measurement instruments

Treatment burden and health-related quality of life were assessed using validated Chinese versions of the following instruments.

#### 2.2.1 Chinese multimorbidity treatment burden questionnaire

Treatment burden was assessed using the Chinese Multimorbidity Treatment Burden Questionnaire (C-MTBQ), a 12-item instrument developed through the translation and cultural adaptation of the original MTBQ (21). Each item is rated on a five-point Likert scale from 0 (not applicable or not difficult) to 4 (extremely difficult). The global score was calculated by multiplying the mean item score by 25, resulting in a standardized score ranging from 0 to 100, with higher scores indicating greater treatment burden. According to the established MTBQ scoring categories, treatment burden was classified as none (0), low (>0 to <10), moderate (10 to <22), or high (≥22). For the primary analysis, the categories were combined into no/low (<10) and moderate/high (≥10) treatment burden. This dichotomization was used to focus the analysis on participants experiencing at least moderate treatment burden and to provide a readily interpretable binary outcome for logistic regression. The cutoff was based on the boundary between the established low and moderate MTBQ categories and was not intended to define a new clinical threshold. In the current sample, the C-MTBQ demonstrated good internal consistency (Cronbach’s *α* = 0.828).

#### 2.2.2 EuroQol Five-Dimension Five-Level Questionnaire

Health-related quality of life was assessed using the EuroQol Five-Dimension Five-Level Questionnaire (EQ-5D-5L) (22). Utility index values were calculated using the Chinese EQ-5D-5L value set and were included in subsequent analyses as a continuous measure of HRQoL.

#### 2.2.3 Sociodemographic, clinical, behavioral, and economic variables

Social demographic, clinical, behavioral and economic information was collected through questionnaires filled out by interviewers. Social demographic characteristics included gender, age, body mass index (BMI), education level, place of residence, marital status and employment status. Age was divided into <70 years old, 70–79 years old and ≥80 years old. BMI was divided into <18.5, 18.5–<24.0 and ≥24.0 kg/m^2^. Education level was divided into primary school or below and junior high school or above. Clinical and medical-related characteristics included the number of chronic diseases, the participation in basic medical insurance for urban and rural residents, and the poverty status of the family. The number of chronic diseases was divided into 2 cases, 3 cases or ≥4 cases. The poverty status was defined based on the original questionnaire or administrative registration. Behavioral variables included current smoking status, current drinking status, frequency of physical exercise and average sleep duration. Current smoking was defined as smoking status, and current drinking was defined as drinking status. Physical exercise was divided into never, 1–2 times, 3–5 times or ≥5 times per week. Sleep duration was divided into <6 h per day, 6–8 h or ≥8 h. Economic variables included annual family income and annual family medical expenditure. Annual family income was divided into <10,000 yuan, 10,000–<30,000 yuan or ≥30,000 yuan, while annual family medical expenditure was divided into <10,000 yuan or ≥10,000 yuan (1, 23). These classifications are based on the responses in the original questionnaire and are made with reference to previous literature. Health-related quality of life (HRQoL) was evaluated using the EQ-5D-5L scale by the above method. Additionally, the EQ VAS was also divided into <80 and ≥80 for descriptive analysis and regression analysis.

For conceptual interpretation, these variables were broadly considered in relation to BOTT domains of treatment-related workload, patient capacity/resources, and health and functional wellbeing; this categorization was interpretive and did not determine the statistical variable-selection procedure. The variables included in this statistical analysis have all been described in the previous text and are listed in Table 1.

**Table 1:** Participant characteristics and univariable comparisons by treatment burden category (*n* = 765).

Variable	Number (*n*/%)		Test statistic	*p*
	No/low treatment burden	Moderate/high treatment burden		
Gender			5.019^a^	0.025
Male	109 (41.6)	168 (33.4)		
Female	153 (58.4)	335 (66.6)		
Age (years)			12.945^a^	0.002
<70	154 (58.8)	240 (47.7)		
70–79	101 (38.5)	224 (44.5)		
≥80	7 (2.7)	39 (7.8)		
BMI (kg/m^2^)			5.684^a^	0.058
<18.5	8 (3.1)	6 (1.2)		
18.5–24.0	80 (30.5)	130 (25.8)		
≥24.0	174 (66.4)	367 (73.0)		
Education			27.372^a^	*p* < 0.001
Primary school or below	129 (49.2)	345 (68.6)		
Junior high school or above	133 (50.8)	158 (31.4)		
Residence			0.066^a^	0.797
Urban area	48 (18.3)	96 (19.1)		
Rural area	214 (81.7)	407 (80.9)		
Marital status			2.590^a^	0.108
Married	227 (86.6)	413 (82.1)		
Not married	35 (13.4)	90 (17.9)		
Employment status			1.841^a^	0.175
Employed	57 (21.8)	89 (17.7)		
Not employed	205 (78.2)	414 (82.3)		
Number of chronic conditions			26.913^a^	*p* < 0.001
2	197 (75.2)	283 (56.3)		
3	43 (16.4)	157 (31.2)		
≥4	22 (8.4)	63 (12.5)		
Enrollment in basic medical insurance for urban and rural residents			16.749^a^	*p* < 0.001
Yes	249 (95.0)	428 (85.1)		
No	13 (5.0)	75 (14.9)		
Household registered as living in poverty			3.945^a^	0.047
Yes	9 (3.4)	35 (7.0)		
No	253 (96.6)	468 (93.0)		
Current smoking status			3.921^a^	0.048
Yes	65 (24.8)	94 (18.7)		
No	197 (75.2)	409 (81.3)		
Current alcohol consumption			5.048^a^	0.025
Yes	80 (30.5)	116 (23.1)		
No	182 (69.5)	387 (76.9)		
Frequency of physical exercise (times/week)			9.531^a^	0.023
Never	48 (18.3)	135 (26.8)		
1–2	39 (14.9)	63 (12.5)		
3–5	54 (20.6)	74 (14.7)		
≥5	121 (46.2)	231 (45.9)		
Sleep duration (h)			6.275^a^	0.043
<6	87 (33.2)	188 (37.4)		
6–8	124 (47.3)	251 (49.9)		
≥8	51 (19.5)	64 (12.7)		
Annual household income (yuan)			14.691^a^	0.001
<10,000	81 (30.9)	227 (45.1)		
<30,000	80 (30.5)	128 (25.4)		
≥30,000	101 (38.5)	148 (29.4)		
Annual household healthcare expenditure (yuan)			10.417^a^	0.001
<10,000	134 (51.1)	196 (39.0)		
≥10,000	128 (48.9)	307 (61.0)		
EQ VAS score			7.855^a^	0.005
<80	120 (45.8)	284 (56.5)		
≥80	142 (54.2)	219 (43.5)		
Health utility index value, median [M(*P*_25_, P_75_)]	0.9 (0.8,1)	0.8 (0.7,0.9)	29.261^b^	*p* < 0.001

a χ^2^ value.

b H value.

BMI, body mass index; EQ VAS, EuroQol Visual Analogue Scale. Data are presented as *n* (%) unless otherwise indicated.

### 2.3 Statistical analysis

The primary analytical focus was the association between HRQoL, assessed using the EQ-5D-5L health utility index, and moderate/high treatment burden, while associations with other sociodemographic, clinical, behavioral, and economic characteristics were considered exploratory. Data were entered and managed using Microsoft Access, and statistical analyses were performed using SPSS version 27.0 and R software. Categorical variables were summarized as frequencies and percentages, and between-group differences were assessed using Pearson’s chi-square test. Continuous variables that were not normally distributed were expressed as medians and interquartile ranges [M (*P*_25_, P_75_)] and compared between the no/low and moderate/high treatment burden groups using the Kruskal–Wallis H test. Treatment burden category was specified as the dependent variable, with no/low treatment burden coded as 0 and moderate/high treatment burden coded as 1. Univariable analyses were first conducted to examine crude differences between the two treatment-burden groups. Given the exploratory nature of the study, variables showing statistically significant associations in the univariable analyses were subsequently entered into the multivariable binary logistic regression model. This data-driven selection strategy was used to obtain a parsimonious exploratory model. Because covariate inclusion was not based on a fully prespecified causal framework, the resulting estimates should be interpreted as adjusted associations within the selected covariate set rather than as estimates from a comprehensive confounder-controlled etiologic model.

### 2.4 Logistic regression and random forest models

Multivariable logistic regression was used to examine adjusted associations between the selected variables and moderate/high treatment burden and to estimate adjusted odds ratios (ORs) with 95% confidence intervals (CIs). A random forest analysis was subsequently conducted as a supplementary exploratory analysis using the variables retained in the multivariable logistic regression model (24–26). The purpose of the random forest analysis was not to independently identify predictors or to develop a clinical prediction model, but to provide additional descriptive information on the relative importance of the selected variables in distinguishing between no/low and moderate/high treatment burden (25, 26). Accordingly, the resulting variable-importance estimates should be interpreted as conditional on the restricted predictor set used in the regression-based analysis. The random forest model included 500 trees, and the number of candidate variables considered at each split was set to the square root of the total number of input variables, consistent with commonly used random forest settings (27, 28). Ten-fold cross-validation was used for internal model assessment. The area under the receiver operating characteristic curve (AUC) was reported as a descriptive measure of discrimination within the available dataset. Variable importance was quantified using the mean decrease in Gini impurity, with larger values indicating a greater contribution to classification within the fitted random forest model (29, 30). Because the model was not externally validated, its discriminatory performance and variable-importance estimates should be interpreted cautiously.

## 3 Results

### 3.1 Participant characteristics

A total of 765 patients with multimorbidity were included in the analysis. Of these, 262 (34.2%) were classified as having no/low treatment burden, whereas 503 (65.8%) were classified as having moderate/high treatment burden. The sample comprised 277 men (36.2%) and 488 women (63.8%). Regarding age, 394 participants (51.5%) were younger than 70 years, 325 (42.5%) were aged 70–79 years, and 46 (6.0%) were aged 80 years or older. Most participants had a body mass index of ≥24.0 kg/m^2^, and 474 (62.0%) had received primary-school education or below. With respect to disease burden, 480 participants (62.7%) had two chronic conditions, 200 (26.1%) had three, and 85 (11.1%) had four or more. Most participants were not current smokers or drinkers. A total of 352 participants reported engaging in physical exercise more than five times per week, while 375 reported sleeping for 6–8 h per day. Regarding economic status, 308 participants (40.3%) had an annual household income (yuan) of <10,000 yuan, 208 (27.2%) had an income of 10,000–<30,000 yuan, and 249 (32.5%) had an income of ≥30,000 yuan. In addition, 435 participants (56.9%) reported annual household healthcare expenditure (yuan)of ≥10,000 yuan. A total of 404 participants (52.8%) had an EQ VAS score of <80, whereas 361 (47.2%) had a score of ≥80 (Table 1).

### 3.2 Univariable analysis of factors associated with treatment burden

Univariable analyses showed that treatment burden category differed significantly according to gender, age, educational attainment, number of chronic conditions, enrollment in the basic medical insurance scheme for urban and rural residents, poverty status, current smoking status, current alcohol consumption, frequency of physical exercise, sleep duration, annual household income (yuan), annual household healthcare expenditure (yuan), self-rated health status, and health utility index value. A higher proportion of moderate/high treatment burden was observed among women, older participants, those with lower educational attainment, those with a greater number of chronic conditions, participants who were not enrolled in the basic medical insurance scheme for urban and rural residents, and those identified as living in poverty. Moderate/high treatment burden was also more common among participants with lower annual household income (yuan), higher annual household healthcare expenditure, poorer self-rated health, and lower health utility index values. Participants who reported no physical exercise or shorter sleep duration also tended to have a higher proportion of moderate/high treatment burden. No statistically significant differences were found according to body mass index, place of residence, marital status, or employment status (all *p* > 0.05) (Table 1).

### 3.3 Multivariable logistic regression analysis of factors associated with treatment burden

Treatment burden category was specified as the dependent variable, with no/low treatment burden coded as 0 and moderate/high treatment burden coded as 1. Variables showing statistically significant associations in the univariable analyses were entered into the multivariable binary logistic regression model. After adjustment for the variables included in the model, educational attainment, number of chronic conditions, current smoking status, frequency of physical exercise, annual household income, annual household healthcare expenditure, and health utility index remained statistically associated with moderate/high treatment burden (all *p* < 0.05) (Table 2).

**Table 2:** Multivariable binary logistic regression analysis of factors associated with moderate/high treatment burden.

Variable	*B*	*S.E*	*Wald*	***p***	*OR* (95%*CI*)
Gender (reference: male)					
Female	−0.254	0.243	1.101	0.294	0.775 (0.482,1.247)
Age (reference:<70 years)					
70–79	0.279	0.188	2.212	0.137	1.322 (0.915,1.911)
≥80	0.684	0.463	2.182	0.140	1.981 (0.800,4.907)
Education (reference: primary school or below)					
Junior high school or above	−0.407	0.198	4.204	0.040	0.666 (0.451,0.982)
Number of chronic conditions (reference: 2)					
3	0.645	0.215	8.994	0.003	1.906 (1.250,2.905)
≥4	−0.025	0.294	0.007	0.931	0.975 (0.548,1.734)
Enrollment in basic medical insurance for urban and rural residents (reference: yes)					
No	0.499	0.346	2.080	0.149	1.647 (0.836,3.244)
Household registered as living in poverty (reference: yes)					
No	0.626	0.466	1.805	0.179	1.871 (0.750,4.665)
Current smoking status (reference: yes)					
No	0.580	0.290	4.002	0.045	1.786 (1.012,3.154)
Current alcohol consumption (reference: yes)					
No	−0.121	0.299	0.163	0.687	0.886 (0.493,1.593)
Frequency of physical exercise (reference: never)					
1–2 times/week	−0.625	0.298	4.394	0.036	0.535 (0.298,0.960)
3–5 times/week	−0.599	0.286	4.382	0.036	0.549 (0.313,0.963)
≥5 times/week	−0.248	0.239	1.079	0.299	0.780 (0.488,1.246)
Sleep duration (reference: <6 h)					
6–8 h	0.187	0.199	0.880	0.348	1.205 (0.816,1.781)
≥8 h	−0.437	0.263	2.752	0.097	0.646 (0.385,1.083)
Annual household income (yuan) (reference: <10,000)					
<30,000	−3.517	0.633	30.886	*p* < 0.001	0.030 (0.009,0.103)
≥30,000	−3.552	0.628	31.993	*p* < 0.001	0.029 (0.008,0.098)
Annual household healthcare expenditure (yuan) (reference: <10,000)					
≥10,000	3.669	0.617	35.34	*p* < 0.001	39.200 (11.694,131.396)
EQ VAS score (reference: <80)					
≥80	−0.244	0.186	1.708	0.191	0.784 (0.544,1.129)
Health utility index value	−1.149	0.562	4.190	0.041	0.317 (0.105,0.952)

OR, odds ratio; CI, confidence interval; SE, standard error; EQ VAS, EuroQol Visual Analogue Scale.

### 3.4 Complementary random forest analysis and variable importance assessment

A random forest analysis was performed as a supplementary exploratory approach using the variables retained in the multivariable logistic regression model. The model yielded an AUC of 0.890 in the internal model assessment. Given the restricted predictor set and the absence of external validation, this value is reported descriptively and should not be interpreted as evidence of validated predictive performance (Figure 2).

**Figure 2:**
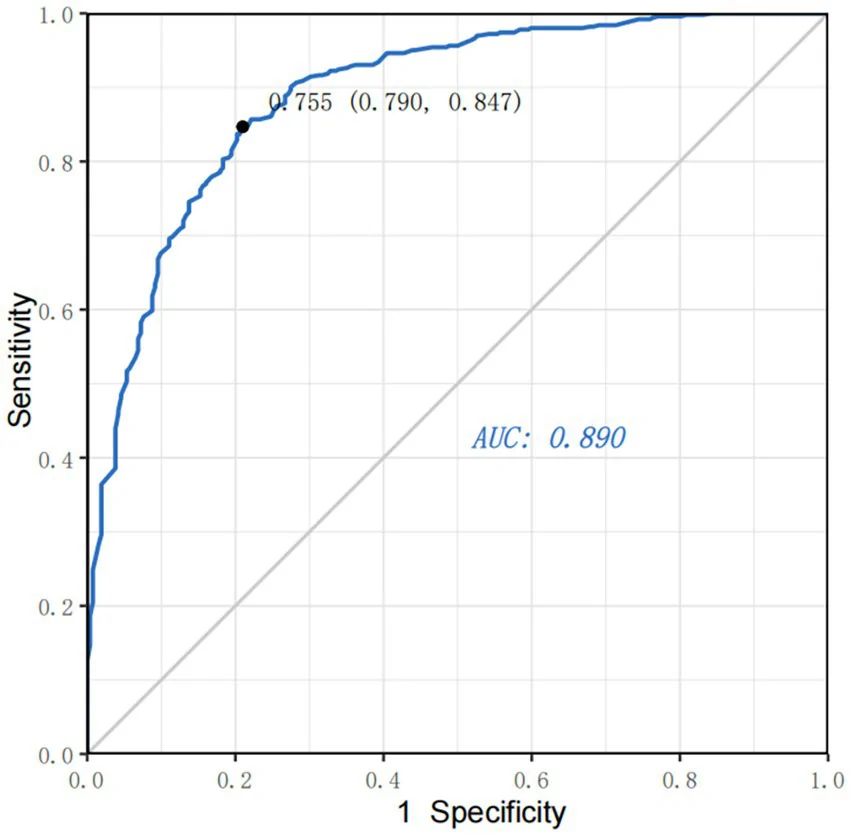
Receiver operating characteristic curve for the random forest model.

Within the variables included in the random forest model, the health utility index showed the largest mean decrease in Gini impurity, followed by annual household income, annual household healthcare expenditure, frequency of physical exercise, and number of chronic conditions. These variable-importance estimates describe the relative contribution of the included variables to classification within this specific model and should be interpreted as conditional on the selected predictor set (Figure 3).

**Figure 3:**
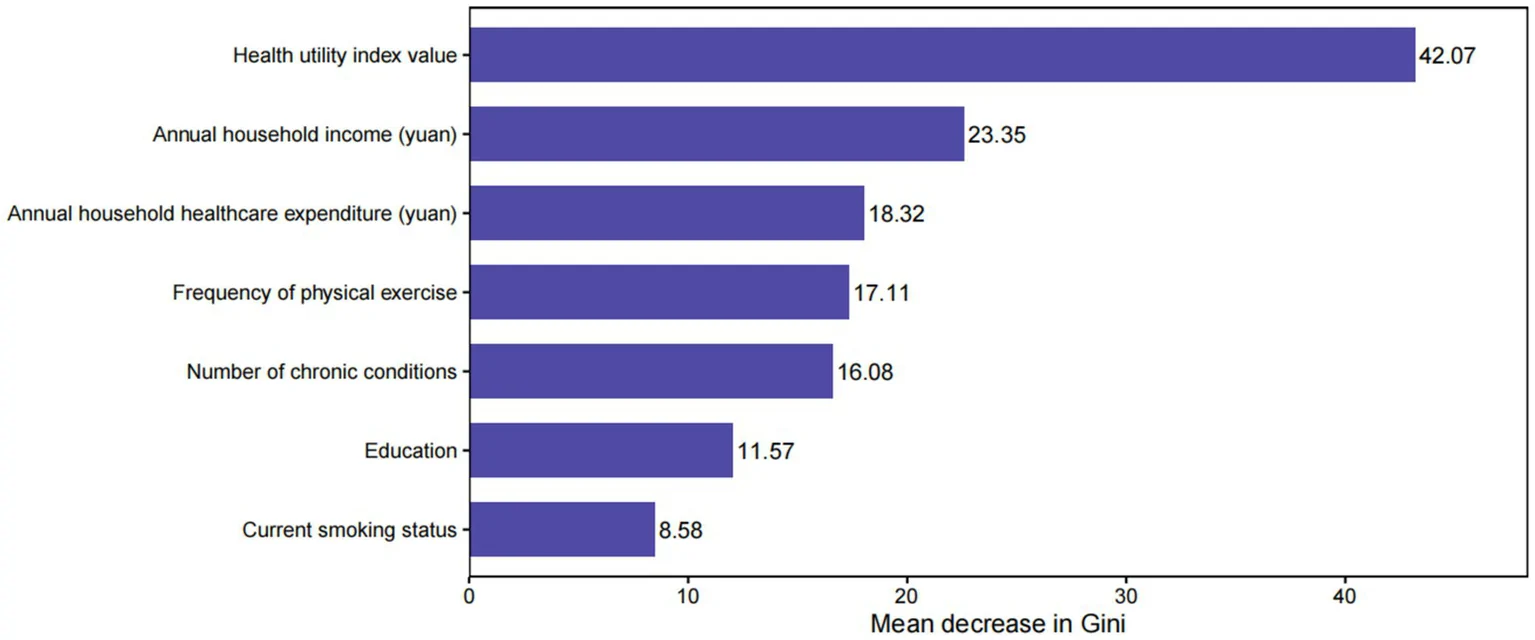
Variable importance ranking based on mean decrease in Gini impurity.

## 4 Discussion

### 4.1 Principal findings and implications

The principal finding was the inverse association between HRQoL and treatment burden. A lower EQ-5D-5L health utility index was independently associated with moderate/high treatment burden and showed the highest relative importance among the variables included in the complementary random forest analysis (15, 31). Socioeconomic circumstances, the number of chronic conditions, educational attainment, and physical activity were also associated with treatment burden. Taken together, the findings suggest that treatment burden in multimorbidity is closely connected not only to treatment workload and economic circumstances but also to patients’ perceived health and functional capacity. This may have practical relevance for primary care, where routine HRQoL assessment could help identify individuals who may be less able to cope with the demands of complex long-term treatment. The present study adds regional evidence by considering HRQoL together with socioeconomic, clinical, and behavioral characteristics within the same analytical framework. Logistic regression provided adjusted association estimates within the selected covariate set, whereas random forest analysis provided supplementary information on relative variable importance. These approaches addressed different aspects of the data and should not be interpreted as independent confirmation of causality or predictive validity.

### 4.2 Household economic circumstances and treatment burden

Multivariable analysis showed that higher household income was associated with lower odds of moderate/high treatment burden, whereas annual household healthcare expenditure of ≥10,000 yuan was associated with markedly higher odds. Both variables also ranked highly in the complementary random forest analysis. This aligns with previous evidence linking multimorbidity to increased costs and financial hardship (32, 33). Treatment burden encompasses not only time and effort but also financial demands (34). Long-term multimorbidity management may involve expenditures related to medications, repeated examinations, healthcare visits, rehabilitation, and other forms of continuing care, which may place additional pressure on household resources (35, 36). However, the adjusted odds ratios for household income and healthcare expenditure were unusually large and should be interpreted with substantial caution. Their magnitude differed considerably from the corresponding univariable patterns, and the confidence interval for healthcare expenditure was wide. These estimates may have been influenced by the categorical specification of the economic variables, uneven distributions across categories, correlations between household income and healthcare expenditure or other clinical characteristics, residual confounding, and instability associated with the data-driven covariate-selection strategy. Because additional diagnostic or sensitivity analyses were not performed, the robustness of the magnitude of these estimates cannot be established. Therefore, the odds ratios should not be interpreted as precise estimates of effect size. A more cautious interpretation is that household economic circumstances were associated with treatment burden within the fitted model, while the magnitude of these associations remains uncertain. From a clinical and health-service perspective, the findings support continued attention to financial pressures experienced by people with multimorbidity. Strategies such as improving financial protection, facilitating access to chronic-disease services, and reducing avoidable treatment-related workload may warrant consideration (37–39). Further studies using more detailed measures of household income, healthcare expenditure, and disease-related costs are needed to clarify these relationships.

### 4.3 Number of chronic conditions and treatment burden

Participants with three chronic conditions had higher odds of moderate/high treatment burden than those with two conditions. In contrast, no statistically significant association was observed among participants with four or more conditions. Therefore, the present findings do not support a simple monotonic or dose–response relationship between the number of chronic conditions and treatment burden.

The cumulative complexity model (40, 41) suggests that treatment burden may arise when treatment and self-management workload exceeds patients’ available capacity (40, 42, 43). However, the number of chronic conditions alone may not adequately capture this complexity. Treatment burden may also vary according to the specific combinations and severity of chronic conditions, medication burden, healthcare utilization, and the compatibility of multiple treatment regimens (44, 45). The absence of a statistically significant association among participants with four or more conditions may also reflect heterogeneity within this group and its relatively small sample size. Accordingly, condition count should be interpreted as only one aspect of multimorbidity rather than as a comprehensive measure of clinical complexity. The included chronic conditions were also clinically heterogeneous and may differ in their treatment workload, functional impact, and effects on HRQoL. Therefore, individuals with the same number of chronic conditions may experience different levels of treatment burden depending on their specific disease combinations. From a care-management perspective, individualized treatment planning, medication review, improved coordination of care, and reduction of avoidable treatment workload may help address the challenges experienced by patients with multimorbidity (46, 47, 53). Future studies should examine specific multimorbidity patterns, disease severity, medication burden, and healthcare utilization to better characterize the relationship between clinical complexity and treatment burden.

### 4.4 Educational attainment and health behaviors are associated with treatment burden

Lower educational attainment and limited health literacy are associated with difficulties in understanding disease information, managing complex regimens, and adhering to treatment, which may increase perceived long-term care demands (48, 49). In contrast, regular physical activity may be associated with better physical and psychological functioning and greater self-management capacity, which could help patients cope more effectively with treatment demands (50). However, some older adults may lack awareness of its benefits, professional guidance, or the ability to sustain regular activity. Therefore, primary healthcare institutions should provide accessible, individualized health education and incorporate exercise prescriptions or community-based programs adapted to each patient’s health status and preferences—carefully designed to support self-management without adding extra workload. These findings align with current national initiatives in China that promote health literacy and self-management through primary care services, reinforcing the importance of tailored educational interventions.

### 4.5 Health-related quality of life is inversely associated with treatment burden

This study found that a higher EQ-5D-5L health utility index was associated with lower odds of moderate/high treatment burden, indicating an inverse association between HRQoL and treatment burden. This relationship is clinically meaningful because the capacity to manage treatment tasks may depend not only on disease complexity and healthcare contacts but also on patients’ physical, psychological, and functional resources, which are reflected in HRQoL measures such as the EQ-5D-5L. The association may also be bidirectional: substantial treatment burden can interfere with daily activities and wellbeing (51), whereas poorer health status may reduce patients’ capacity to complete complex treatment and self-management tasks (52). Because the present study is cross-sectional, the direction of this relationship cannot be determined and causal interpretations should be avoided. Nevertheless, the finding is consistent with previous evidence linking higher treatment burden with poorer HRQoL among people with multimorbidity (15). Within the restricted set of variables included in the supplementary random forest analysis, the health utility index showed the largest mean decrease in Gini impurity. This finding indicates its relatively strong contribution to classification within the selected predictor set; it should not be interpreted as evidence that HRQoL is the most important determinant of treatment burden or as independent confirmation of the regression findings. HRQoL may provide useful supplementary information when assessing treatment burden, although its relative importance remains conditional on the variables analyzed. In contrast to mechanism-oriented research examining the role of psychological and social resources in the relationship between treatment burden and HRQoL, the present study considered HRQoL alongside a broader set of characteristics associated with treatment burden.

## 5 Limitations

The study was conducted in Shandong Province. Although the sample covered areas with different geographic and socioeconomic characteristics, regional differences in economic conditions and healthcare may limit the applicability of the findings elsewhere in China. We could not compare included and excluded respondents because detailed information on the latter was unavailable, leaving potential selection bias unassessed. The exclusion of people with severe psychiatric, intellectual, cognitive, or communication impairment may also have introduced selection bias and limits the applicability of the findings to more vulnerable adults with multimorbidity. Several variables were self-reported and may be affected by recall or social desirability bias. The chronic-condition list was drawn from the original survey and did not fully match standardized multimorbidity lists, which limits comparisons with other studies. Stroke and cerebral infarction may overlap, but the analysis followed the original survey coding without reclassification. This may have affected the number of conditions recorded for some participants. Using an unweighted condition count, without examining disease-specific severity, disease clusters, or sex-specific patterns, also limited our ability to account for differences in multimorbidity profiles. The cross-sectional design prevents conclusions about temporal sequence or causality, particularly in the relationship between HRQoL and treatment burden. Covariates were selected according to univariable statistical significance rather than a prespecified causal framework. This approach may have omitted relevant confounders and affected the stability of the estimates. The regression findings should therefore be interpreted as exploratory associations adjusted for the included covariates. Combining treatment burden into two groups reduced the information available from the continuous MTBQ score and its original four categories and may have influenced the associations observed. Analyses using continuous scores or ordered categories could help assess this in future research. The random forest analysis included only variables retained in the regression model, limiting its independent exploratory value. Its importance rankings depend on this selected set of variables. The reported AUC should be interpreted as an internal estimate of discrimination and does not establish predictive performance in other populations. Further research should examine a broader range of candidate predictors and validate the model using independent data.

## 6 Conclusion

This study found that treatment burden among adults with multimorbidity was associated with HRQoL, socioeconomic circumstances, health behaviors, and the number of chronic conditions. With respect to multimorbidity count, participants with three chronic conditions had higher odds of moderate/high treatment burden than those with two conditions, whereas no statistically significant association was observed for those with four or more conditions. Therefore, the observed pattern does not support a simple dose–response relationship between condition count and treatment burden. A lower EQ-5D-5L health utility index remained associated with moderate/high treatment burden after adjustment for the variables included in the regression model. Within the restricted predictor set used in the supplementary random forest analysis, health utility also contributed relatively strongly to classification. These findings suggest that HRQoL may provide useful supplementary information when assessing treatment burden among adults with multimorbidity. However, all findings should be interpreted as cross-sectional associations. Temporal direction and causal relationships cannot be established, and the observed associations require confirmation in longitudinal and external datasets.

## Data Availability

The original contributions presented in the study are included in the article/supplementary material, further inquiries can be directed to the corresponding authors.
